# Freshwater Sponge *Tubella variabilis* Presents Richer Microbiota Than Marine Sponge Species

**DOI:** 10.3389/fmicb.2019.02799

**Published:** 2019-12-03

**Authors:** Marinella Silva Laport, Ulisses Pinheiro, Caio Tavora Coelho da Costa Rachid

**Affiliations:** ^1^Institute of Microbiology Paulo de Góes, Federal University of Rio de Janeiro, Rio de Janeiro, Brazil; ^2^Department of Zoology, Federal University of Pernambuco, Recife, Brazil

**Keywords:** bioactive compounds, culture-dependent approach, global sponge-microbiome, sponge-associated bacteria, *Spongillida*

## Abstract

Sponges can host diverse and abundant communities of microorganisms, which constitute an interesting source of bioactive compounds. Thus, to broaden our knowledge about the diversity of the microbiota that is found in freshwater sponges, the microbial community of *Tubella variabilis* was analyzed using culture-independent and culture-dependent approaches. Additionally, sponge-associated bacteria were compared with those living in the surrounding waters. Bacteria were also tested for antimicrobial production. Overall, the microbial composition identified comprises at least 44 phyla belonging mainly to Proteobacteria and low percentages of Bacteroidetes, Acidobacteria, and Verrucomicrobia. Alphaproteobacteria was the dominant class in *T. variabilis* while Betaproteobacteria was dominant in freshwater. Our data also revealed a high richness of bacteria in comparison to another freshwater sponge and 32 marine sponges. A global comparison of the structure of microbiota of different sponges showed that the main structuring factor may be the sponge environment, with *T. variabilis* and all freshwater sponges clustering together, and far away from the marine sponges. Bacterial strains from sponges and from freshwater were isolated and 163 morphotypes were phylogenetically identified. These belong to 26 genera, of which 12 were exclusively found in sponge samples and three only in freshwater. Inhibitory activities were also detected among 20–25% of the isolates from sponges and freshwater, respectively. This study presents new information on the composition of the microbial community found in freshwater sponges, which is diverse, abundant and distinct from some marine sponges. Moreover, the antimicrobial activity observed from the bacterial strains might play an important role in shaping microbial communities of the environment.

## Introduction

Porifera is a phylum comprising filter-feeding and sessile animals that play important ecological roles for benthic fauna around the world (van Soest et al., 2012) and consists of four classes: Calcarea, Demospongiae, Hexactinellida, and Homoscleromorpha (Gazave et al., 2012). Although a vast majority of sponge species can be found in marine environments (9,162 valid species), there are also 248 species living in freshwaters worldwide listed in the World Porifera database (van Soest et al., 2019). These ancient invertebrates can be found in many different continental aquatic environments such as streams, rivers, lakes, ponds, and caves. With the exception of Antarctica, freshwater sponges are spread throughout all biogeographic regions. They contribute to benthic primary productivity in lake and stream ecosystems (Manconi and Pronzato, 2008; Gazave et al., 2012; van Soest et al., 2012, 2019). Freshwater sponges are able to live in many adverse environmental conditions and colonize a wide variety of habitats with a hard substrate such as rocks, shells of mollusks, wood debris, roots, branches in riparian zone and macrophytes (Manconi and Pronzato, 2008).

All freshwater sponges belong to the class Demospongiae and to the order *Spongillida*, currently consisting of 63 genera with many endemic species (van Soest et al., 2019). These sponges are found as thick or thin crusts, tree-shaped branches or cloddy bulks, and are found on aquatic plants, bivalves or gastropods, stones, wood, and various anthropogenic substrata such as cement, glass, and metal (Manconi and Pronzato, 2008). *Tubella variabilis* (Bonetto and Ezcurra de Drago, 1973) is an encrusting thin freshwater sponge. It is beige and green and the consistency of live sponges can vary from fragile to moderately soft (Nicacio and Pinheiro, 2015).

The association between bacteria and sponges is one of the oldest that has been reported between microorganisms and metazoa, and probably exists since the pre-Cambrian period (over 600 million years ago) (Wilkinson et al., 1984). The sponges host a complex, rich and abundant microbial community, which most likely assists in evolution, ecology and health of the host (Taylor et al., 2007; Webster and Taylor, 2012).

The relationship between sponges and their associated microorganisms are so important that bacteria may constitute up to 38% of the sponge mass (Vacelet, 1975) and can participate in different functions such as host defense, as nutrient source and turnover of metabolites (Taylor et al., 2007; Santos-Gandelman et al., 2014). The evolutionary and ecological success of symbiosis between bacteria and sponges is mirrored by their enormous biotechnological potential: sponges have been highlighted in the Animalia kingdom as sources of several bioactive compounds, and, in some cases, the true producer are the sponge-associated bacteria (Santos-Gandelman et al., 2014). The vast majority of these studies involve only marine sponges, because they are more attractive in terms of their distribution, diversity and abundance all over the globe when compared to freshwater sponges (van Soest et al., 2012). However, information about freshwater sponge-associated bacteria is still rudimentary and the role of symbiotic bacteria in the ecology, nutrient turnover and host defense is a mystery to be unraveled (Gernert et al., 2005; Costa et al., 2012; Kaluzhnaya et al., 2012; Keller-Costa et al., 2014; Gaikwad et al., 2016).

The use of Next Generation Sequencing (NGS) technology, is greatly improving our knowledge of host-microbiota interactions, including sponge associated-bacteria. Thomas et al. (2016) analyzed the composition and diversity of 81 different Porifera species, which showed great diversity and variability of the sponge microbiome. Nevertheless, the mentioned work assessed only marine sponges and studies on the microbial diversity of the freshwater sponges remains scarce. Thus, considering their important role in freshwater systems, studies on the microbiota of freshwater sponges are of great value to science.

In the present study, we evaluated the bacterial community associated with the *T. variabilis*, a Brazilian freshwater sponge and compared its structure with the microbiota of the surrounding freshwater and with that of other freshwater and marine sponges. Analyses were performed using culture-independent and culture-dependent techniques. Additionally, a probable role of production of antimicrobials within the bacterial communities in the freshwater environment was also addressed.

## Materials and Methods

### Site Description and Sampling

Twelve samples of *T. variabilis* were collected, consisting of four fragments (numbered from 1 to 4) of different parts of three distinct sponge individuals (called A, B, C). The sponges were found at a distance of 5 m from each other at a depth of 5–20 cm. Two of them were collected from a stream environment and one from a standing freshwater environment. Collections were made manually in the artificial channel (8°1′9.40″S, 34°56′39.93″W) that provides water to fish farm tanks of Universidade Federal Rural de Pernambuco with water from the da Prata River in the city of Recife (Pernambuco, NE Brazil). Specimens were deposited at the Porifera Collections of Universidade Federal de Pernambuco, Brazil, UFPEPOR, under the numbers 2205, 2206, and 2207. This collection was supported by a permanent license for the collection of zoological material under the number 18100-1, issued by the System of Authorization and Information on Biodiversity (*Sistema de Autorização e Informação em Biodiversidade* – SISBIO) of the Ministry of Environment (*Ministério do Meio Ambiente* – MMA), Brazil.

The fragments (1–3) of each sponge (A–C) were stored for cultivation-dependent analysis as follows: samples A1, A2, A3, B1, B2, B3, C1, C2, and C3 were placed separately in Falcon tubes containing 25 ml of sterile freshwater, previously collected in the respective sites, supplemented with 1 μg/ml of amphotericin B (antifungal drug) (Sigma-Aldrich). Regarding cultivation-independent analysis, the remaining fragments A4, B4, and C4 were placed in CHAOS solution (guanidine thiocyanate 4 M, N-lauryl sarcosil 0.5%, Tris 25 mM pH 8.0, 2-mercaptoethanol 0.1 M). All samples were kept on ice and taken to the laboratory within 24 h.

Each sponge sample destined for cultivation analysis was ground using mortar and pestle and vortexed for 1 min. All tubes were allowed to stand for 1 min, and from the resulting supernatant, serial 10-fold dilutions were plated (in duplicate) on the following solid culture media (Difco): BHI, 10-fold diluted BHI, R2A, Malt, and Czapek-Dox. All media were supplemented with 1.5% agar and 1 μg/ml amphotericin B.

Surrounding freshwater samples from the same three sites were also collected in duplicate (called WA_1__–__2_, WB_1__–__2_, WC_1__–__2_) and filtered through a 0.22 μm pore size filter. One filter (WA-C_1_) was placed in CHAOS solution for molecular analysis and the second (WA-C_2_) was washed with 1 ml of sterile freshwater, serial 10-fold dilutions were performed and plated as explained above.

### DNA Extraction and Sequencing

The genomic DNA of all three distinct sponge individuals (A4, B4, and C4 samples) and freshwater (WA_1_, WB_1_, and WC_1_ samples) was extracted using a modified phenol-chloroform protocol (Fukami et al., 2004). The quality of extracted DNA was checked by 0.8% agarose gel electrophoresis and its quantity determined by using a NanoVue Plus spectrophotometer (GE Healthcare Life Sciences). DNA was stored at −20°C until use for PCR amplification.

PCR amplifications were carried out on sponge genomic DNA targeting the mitochondrial gene cytochrome oxidase subunit I (*cox-1*) with 10 pmol of each primer LCO1490 and HCO2189 (Folmer et al., 1994), 1 × buffer GO TAQ G2 Green Master Mix (Promega), 0.4 mg/ml of BSA (Sigma-Aldrich), and 0.05% of Igepal (Sigma-Aldrich) in a 25 μL volume.

Amplification consisted of pre-denaturation for 3 min at 95°C, followed by 36 cycles of 30 s at 95°C, 45 s at 43°C, and 1 min at 72°C, and a final extension step for 10 min at 72°C. The amplicons (∼660 bp) were checked by 2% agarose gel electrophoresis using Low DNA Mass Ladder (Thermo Fisher Scientific) as standard. PCR products were sequenced by the Sanger method in ABI 3500 automated sequencers (Biotecnologia, Pesquisa e Inovação – BPI, SP, Brazil).

The sequences generated from *cox-1* had their quality inspected and edited with the Sequence Scanner 2 software (Thermo Fisher Scientific). The resulting sequences were submitted to the National Center for Biotechnology Information database (NCBI).

### Bacterial Community Analysis

#### DNA Amplification, Library Preparation

Total DNA from sponge and freshwater samples were sent to the BPI sequencing service. Sponge and freshwater DNA were purified with magnetic beads prior to performing the PCRs. DNA was PCR amplified in triplicate. Reactions contained 0.3 μM of each universal primer for V4 regi on of 16S rRNA (Caporaso et al., 2011), 1 × buffer GoTaq Colorless Master Mix (Promega), 20 ng of genomic DNA in a total volume of 20 μl. Amplification was performed using the following program: 94°C for 3 min, then 29 cycles of 94°C for 45 s, 50°C for 1 min, and 72°C for 1 min, and a final extension of 72°C for 1 min 30 s. Amplicons were analyzed using 2% agarose gel electrophoresis. After combining the triplicate products, they purified using Agencourt AMPure XP kit (Thermo Fisher Scientific) and quantified by real-time PCR, all according to the manufacturer’s protocol KAPA-KK4824 (Library Quantification Kit – Illumina/Universal, Kapa Biosystems). An equimolar pool of DNA was generated by normalizing all samples at 2 nM for the sequencing, which was conducted using the Illumina MiSeq new generation sequencing system (Illumina^®^ Sequencing). After sequencing, a FASTQ file containing the sequences was generated.

#### Sequence Identification and Bioinformatics Analysis

The raw joined sequences were processed using the Mothur v.1.39.1 software (Schloss et al., 2009). The paired raw sequences were joined into contigs with make.contig command and screened using screen.seqs with the following parameter: maxambig = 0, maxlength = 275, minlength = 220. The sequences were then aligned using a modified Silva database (passed by a virtual PCR with the same primers of the samples) as reference (Quast et al., 2013) and the resultant alignment was submitted to screen.seqs and filter.seqs to remove sequences with either bad alignment or uninformative columns of alignment. The sequences were then pre-clustered using the command pre.cluster with parameter diffs = 2. The chimeras were detected with the command chimera.vsearch and then eliminated. Sequences were classified using the classify.seqs command, with the RDP database (Cole et al., 2009) as a reference and a bootstrap cutoff of 80. Sequences classified into chloroplasts, mitochondria, Eukarya, Archaea and those not assigned to any kingdom were removed. The resultant sequences were used as an input for the dist.seqs command. Finally, the sequences were clustered into operational taxonomic units (OTUs), with a cutoff of 3% of dissimilarity, and all singletons were removed. To avoid bias due to sampling effort, the samples were randomly normalized to the same number of sequences (97,945). The taxonomic summary was used to analyze the bacterial composition of each sample. The diversity of each sample was calculated using summary.seqs command. Diversity was evaluated using Shannon Index (Shannon, 1948) and the number of OTUs was used as a measure of sample richness. OTU distribution was used to establish the relationship between samples, and to evaluate significant differences in specific OTUs among water and sponge microbial communities.

#### Nucleotide Sequence Accession Numbers

The data generated were deposited in the NCBI Sequence Read Archive (SRA) and are available under accession number SRP115997.

### Global Sponge-Associated Bacteria Composition Analysis

To understand how the microbiome of *Tubella* compares with other sponge’s microbiomes, a global analysis was run with two other freshwater sponges and 32 different marine sponges. To perform the analysis, we retrieved from SRA and MG-RAST databases the 16S rRNA sequences of 99 samples. The list of sponges used and their respective access numbers are summarized in Supplementary Table S1.

After downloading the sequences, all FASTA files were merged using Mothur v.1.39.1 and then aligned as previously described. Global analysis was possible since all samples were amplified using the same primers for the V4 region of the 16S rRNA. However, the marine sponge samples have a shorter 16S rRNA fragment due to the technology employed during the sequencing (Illumina HiSeq2500). Therefore, all samples were trimmed to the overlapping region after alignment. Sequences were then pre-clustered using the command pre.cluster with parameter diffs = 1. Chimeras were detected with the command chimera.vsearch and then eliminated. Sequences were classified using the classify.seqs command, with the RDP database (Cole et al., 2009) as a reference and a bootstrap cutoff of 80. Sequences classified into chloroplasts, mitochondria, Eukarya, Archaea and those not assigned to any kingdom were removed. The resultant sequences were used as an input for the dist.seqs command. Finally, the sequences were clustered into OTUs, with a cutoff of 3% of dissimilarity, and all singletons were removed. To avoid bias due to sampling effort, the samples were randomly normalized to the same number of sequences (24,600). The OTU distribution was used to calculate the diversity index as previously explained, and to establish the relationship between samples.

### Statistical Analysis

Statistical differences in the diversity and richness indexes, as well as those in the relative abundance of the top thirty OTUs were tested using *t*-test, after testing for normality in PAST 3.11 (Hammer et al., 2001). The relationship of the microbial structure among different samples was assessed using non-metric multidimensional scaling, with Bray–Curtis distance.

### Isolation and Cultivation of Bacteria

Bacterial cultures were incubated for up to 7 days at room temperature (RT, 25 ± 2°C) and examined daily for growth and colony morphology. Bacteria were purified from the primary culture and kept in slant cultures at −20°C. At least three colony-forming units (CFU) of each morphotype from each culture medium were selected based on size, colony appearance, and presence of pigments, as an attempt to cover all the colony morphologies observed. The strains were named with the initial letter of the culture medium, the letter and number of the sponge sample, followed by the order of isolation of CFU from the culture medium. For example, strain BA2-1 was the first strain isolated from fragment 2 of the sponge specimen A on BHI-agar (Santos-Gandelman et al., 2014).

### 16S rRNA Sequence Analysis of Bacterial Isolates

Bacterial DNA was recovered by a thermal lysis protocol consisting of resuspending cellular material from each colony in 25 μl sterile PCR grade water and boiling the suspension at 100^°^C for 15 min. PCR amplification was performed by adding 1 μl of DNA solution to 24 μl containing 1 × buffer GO TAQ G2 Green Master Mix (Promega), 0.4 mg/ml of BSA (Sigma-Aldrich), 0.05% of Igepal (Sigma-Aldrich), and 10 pmol of each universal primer, 27F (5′-GAGTTTGATCMTGGCTCAG-3′) and 1492R (5′-TACGGYTACCTTGTTACGACTT-3′) (Weisburg et al., 1991). Cycle conditions consisted of an initial denaturation step at 94°C for 6 min, followed by 30 cycles at 94°C for 30 s, 55°C for 1 min 30 s and 72°C for 2 min 30 s, and a final elongation step at 72°C for 5 min.

PCR products were analyzed by electrophoresis on a 0.8% agarose gel, purified using the QIAquick PCR Purification Kit (Qiagen), and sequenced using the universal primer 338F (5′-ACTCCTACGGGAGGCAGC-3′) at BPI sequencing service. 16S rRNA gene sequences obtained for the isolates were aligned and classified using the online portal of the SILVA SINA alignment service of the ARB-Silva database^1^ (Pruesse et al., 2007).

### Production of Antimicrobial Substances

Bacterial strains from sponges were screened in triplicate for inhibitory activity employing an antimicrobial substance production assay previously described (Marinho et al., 2009). Hereafter, bacterial strains which tested positive for production of antimicrobial substances were described as “producer (or active)” strains, whereas bacteria used as targets were described as “indicator (or inhibited)” strains. Briefly, 10^7^ cells of each producer strain were spotted onto BHI-agar and incubated at 25°C until the colony diameter reached 5–8 mm. In parallel, *Staphylococcus aureus* ATCC29213 (indicator strain) was grown in BHI broth at 37°C for 18 h. Then 10^5^ cells of the latter mixed with 3 ml of BHI soft agar were poured over the plates. The plates were incubated at 37°C for 24 h and the diameter of the inhibition zone around the spotted strain was measured. An indicator strain was considered sensitive to the activity of the producer strain when it exhibited a clear inhibition zone (and was then considered “inhibited”).

## Results

### Molecular Taxonomy of *T. variabilis*

The *cox-1* sequences of *T. variabilis* analyzed were 99–100% identical to each other at the nucleotide level and were submitted to NCBI database under the accession numbers MG099655, MG099656, and MG099657. For all three sponges, the closest hit from BLAST analysis was *Trochospongilla pennsylvanica* (synonymy *Tubella pennsylvanica*) cytochrome *c* oxidase subunit I, with 98, 89% identity (DQ087503.1). No other *Tubella* sp. *cox-1* gene sequences were publicly available at the time of this study.

### Culture-Independent Analysis

The microbiome analysis revealed agreater richness (evaluated by number of OTU, *p* = 0.02) in *T. variabilis* when compared to the surrounding water. The former showed OTU numbers ranging from 3,762 to 4,709 and the latter ranged from 3,419 to 3,522 (Supplementary Figure S1a). The diversity was also higher in *T. variabilis* (evaluated by Shannon index, *p* = 0.04), with average values of 5.14, compared to 4.3 from freshwater (Supplementary Figure S1b). Rarefaction curves (Supplementary Figure S2) confirmed the higher diversity found in *T. variabilis*.

**FIGURE 1:**
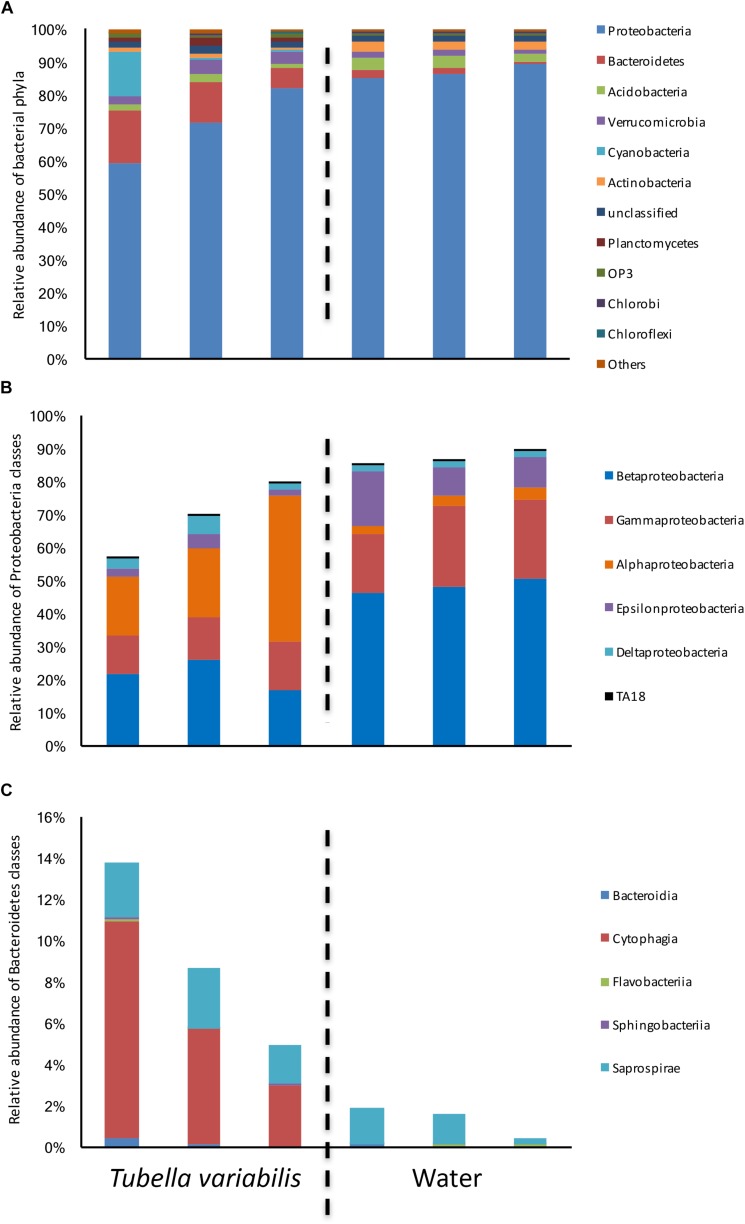
Relative abundance of bacterial groups found in *Tubella variabilis* and surrounding freshwater, obtained by 16S rRNA gene sequencing and classified using RDP database. **(A)** Relative abundance of bacterial phyla. **(B)** Relative abundance of Proteobacteria classes. **(C)** Relative abundance of Bacteroidetes classes.

The bacterial composition at phylum level was dominated by Proteobacteria, which ranged from 60 to 82% in *T. variabilis* and from 85 to 89% in freshwater (Figure 1A), Bacteroidetes, Acidobacteria, Verrucomicrobia, Cyanobacteria and a further 39 phyla and candidate phyla were observed in minor abundance.

Many differences were observed at class level. Alphaproteobacteria was the dominant class in *T. variabilis* while Betaproteobacteria was dominant in freshwater. However, differences in all other classes of Proteobacteria could be observed (Figure 1B). The phylum Bacteroidetes was significantly enriched in *T. variabilis* when compared to freshwater (*p* = 0.03), especially due to the amount of the Cytophagia class which was a hundred times higher in *T. variabilis* than in freshwater (Figure 1C).

The relationship between the structures of microbial communities isolated from *T. variabilis* and freshwater was assessed by using a non-metric multidimensional scaling (Supplementary Figure S3) and revealed a clear separation of the samples based on microhabitat of origin along the horizontal axis. It also showed higher inner variation in *T. variabilis* samples compared to freshwater. However, the community structure was not significantly different between the two environments using Anosim or Permanova (both *p* = 0.10).

**FIGURE 2:**
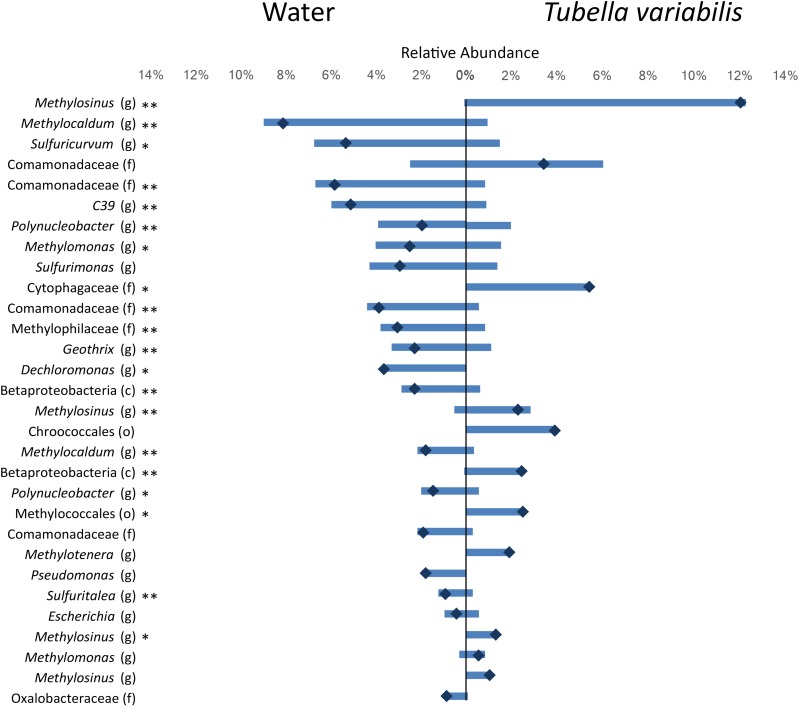
Relative abundance of the top 30 most abundant OTUs (3% dissimilarity cutoff) found in the surrounding freshwater and *T. variabilis.* Each bar represents the average relative abundance of a given OTU. The dark blue mark represents the difference between the abundance of both environments. ^∗^*p* < 0.05, ^∗∗^*p* < 0.01 (*t*-test).

To understand which members of the microbial community were differently distributed between sponge and surrounding water, we identified and tested the difference in the relative abundance of the top thirty most abundant OTUs (which summed in average to 64% of the whole community abundance) (Figure 2). From the top thirty OTUs, 20 showed significant differences between the two environments, and most of those were very abundant in one of them and almost absent in the other. The main OTUs enriched in freshwater belonged to genera *Methylocaldum*, *Sulfuricurvum* and genus C39 (order Rhodocyclales), and family *Comamonadacea*, whereas the main OTUs enriched in *T. variabilis*, belonged to genus *Methylosinus*, and family *Cytophagacea*.

### Analysis of Global Sponge Microbiome Composition

**FIGURE 3:**
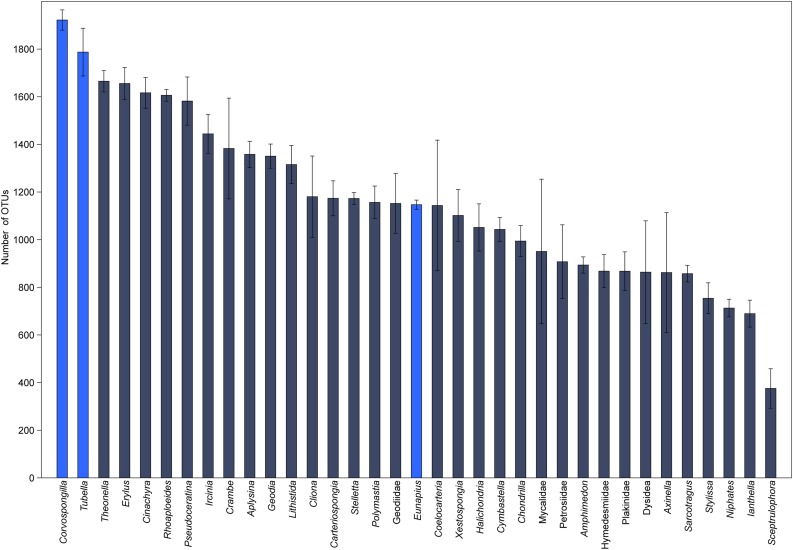
Global richness comparison of the microbial community of *T. variabilis* with two freshwater sponges and 32 marine sponges. The bars represent the average number of OTUs (*n* = 3 or 2 per sponge) with the standard deviation. Bright blue bars are freshwater sponges and in dark blue bars are marine sponges.

**FIGURE 4:**
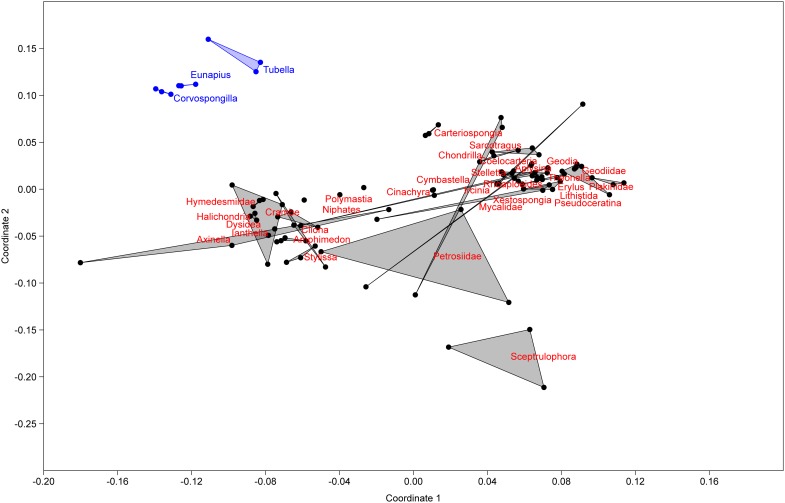
Non-metric multidimensional scaling based on OTU distribution of *T. variabilis* with two freshwater sponges and 32 marine sponges. Freshwater sponges are represented in blue dots and marine sponges in black dots. Ordination stress was 0.18 in the scale of 0–1.

In order to understand their relation, the richness and structure of the microbial community of *T. variabilis* was compared to that of two other freshwater sponges (*Eunapius carteri* and *Corvospongilla lapidosa*) and 32 different marine sponges. The comparison showed an interesting fact regarding richness, with the two richest communities (measured by number of OTUs) being related to freshwater sponges (Figure 3). Additionally, the grouping pattern revealed by NMDS suggests that the type of aquatic ecosystems is the main driving force that shapes the community structure since all freshwater sponges grouped in the top left of the ordination and most of marine sponges grouped in the center of the ordination. The marine sponge *Sceptrulophora* was very distinct from all other sponges, since it did not group near any one of the other sponges due the lower bacterial richness (Figure 4).

### Phylogenetic Affiliation of Isolates

Culture-based analyses revealed a high proportion of mucoid and pigmented CFU among the 453 strains isolated on five different growth media. Among the 303 CFU chosen from *T. variabilis*, 75 strains (24.8%) grew on 10-fold diluted BHI agar, 67 strains (22.1%) on Czapek-Dox agar, 65 strains (21.4%) on R2 agar, 51 strains (16.8%) on BHI agar, and 45 strains (14.9%) on Malt agar. Bacteria were isolated from the different sponges in balanced proportions. The freshwater samples yielded 150 bacterial isolates, of which 38 strains (25.3%) grew on R2 agar, 34 strains (22.7%) on BHI agar, 34 strains (22.7%) on 10-fold diluted BHI agar, 28 strains (18.7%) on Malt agar and 16 strains (10.7%) on Czapek-Dox agar.

Considering the morphotype of each isolate, 163 strains were selected and identified based on 16S rRNA gene sequence analysis. Sequence data were deposited in GenBank database under the accession numbers MH424474.1-MH424486.1, MH426850.1-MH426928.1, MH454615.1-MH454640.1, MH470-383.1-MH470403.1, MH477670.1-MH477693.1.

**FIGURE 5:**
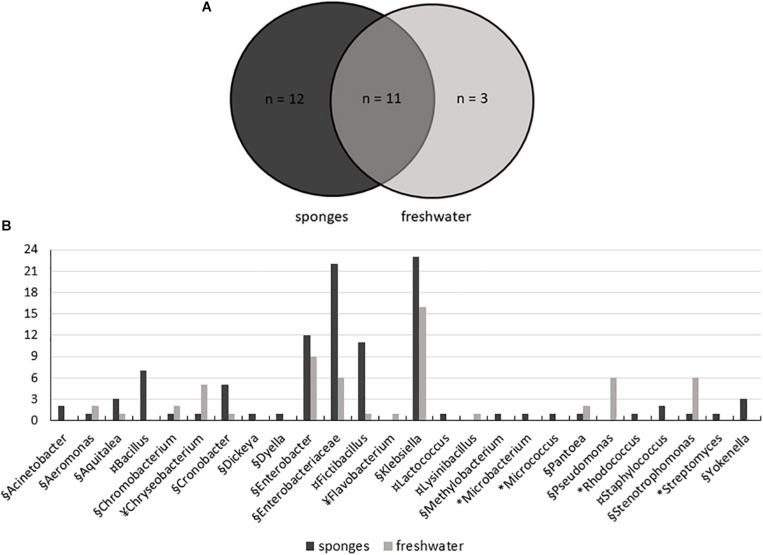
Distribution of bacteria isolated from *T. variabilis* and surrounding freshwater samples. **(A)** Venn diagram shows the numbers of common and unique bacterial strains isolated from sponges and from freshwater. **(B)** Phylogenetic composition of the bacteria culturable community from the *T. variabilis* and freshwater samples. Stacked column bar graph depicting the absolute abundances of bacterial genera and family isolated. Bacterial isolates belonging to Actinobacteria (^∗^), Bacteroidetes (¥), Firmicutes (¤), and Proteobacteria (§) are indicated.

The use of five different culture media contributed to the isolation of high diversity of bacteria, with 104 isolates obtained from sponges and 59 isolates from freshwater samples. A total of 26 genera were represented: 15 genera of Proteobacteria, including (Alpha-, Beta-, Delta-, and Gammaproteobacteria), five genera from the Firmicutes, four genera from the Actinobacteria, and two genera of Bacteroidetes. Twenty-three genera were recovered from *T. variabilis* and 14 from freshwater samples, 12 genera were isolated only from the sponges and three exclusively from water (Figure 5A).

Gammaproteobacteria was the main class identified among the strains, and was predominately affiliated with the *Enterobacteriaceae* family and belonged to the genera *Klebsiella* and *Enterobacter*. Besides enterobacteria, *Pseudomonas* was the most frequently found genus in freshwater samples. Betaproteobacteria and Deltaproteobacteria isolates were recovered from both the sponge and freshwater samples. Finally, a single culture of Alphaproteobacteria belonged to the genus *Methylobacterium* and was isolated from a sponge sample (Figure 5B).

Firmicutes was the second most frequently isolated bacterial phylum, with the predominant genera being *Fictibacillus* and *Bacillus*. *Bacillus* was isolated only from *T. variabilis* samples. The strains of Actinobacteria were also exclusively isolated from sponges and were dominated by single isolates of the *Microbacterium, Micrococcus, Rhodococcus* and *Streptomyces*. All Bacteroidetes belong to the class Flavobacteria of the genera *Chryseobacterium* and *Flavobacterium* (Figure 5B).

### Overlap of Culture-Independent and Culture-Dependent Analysis

**FIGURE 6:**
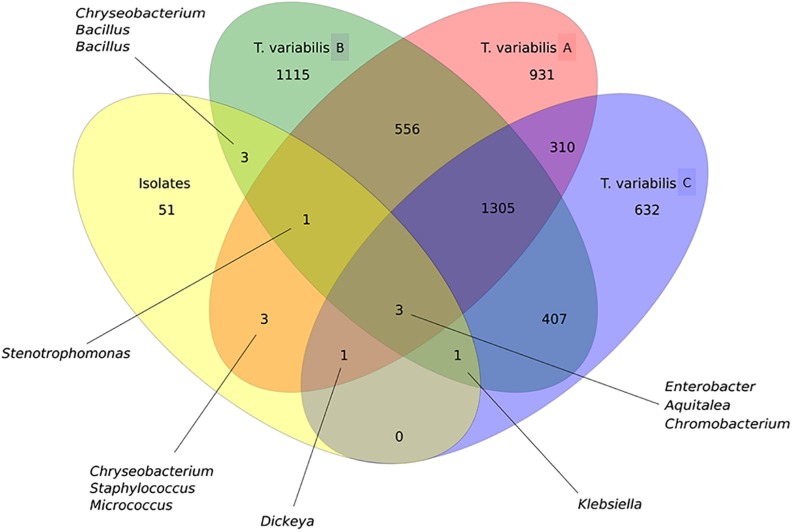
Venn diagram that illustrates the relationship between OTUs that were detected in the *T. variabilis* samples by culture-independent and culture-dependent analysis. A comparison of the overlap in terms OTUs in the three different sponge samples (*T. variabilis* A, B, and C) and their cultivable fractions (isolates). The numbers of OTUs from and shared by each sample are represented inside the oval shapes and the main bacterial genera are indicated. Venn diagram of OTUs at distance 0.03.

In general, OTU sequences obtained from culture-independent analysis overlap much more among each other than with OTUs obtained from the sequences of the isolates. There were 1,305 shared OTUs among the three sponge samples analyzed by NGS (Figure 6).

Proteobacteria was the main phylum isolated and identified among the isolates, as observed in the NGS analysis. However, the relative abundance of the Proteobacteria classes between the two methodologies did not follow the same pattern. There was a predominance of Alphaproteobacteria and Betaproteobacteria in the NGS analysis, whereas bacteria recovered from the culture media showed a greater abundance of Gammaproteobacteria followed by Betaproteobacteria isolates. Although there were only three shared OTUs among all cultured and non-cultured bacteria, they belonged to the genera *Aquitelea* (Betaproteobacteria), *Chromobacterium* (Betaproteobacteria) and *Enterobacter* (Gammaproteobacteria) (Figure 6).

However, if we take a closer look at the overlapping sequences between isolates and NGS data from sample A of *T. variabilis*, eight OTUs were common and were identified as *Aquitalea*, *Chromobacterium*, *Chryseobacterium*, *Dickeya*, *Enterobacter*, *Micrococcus*, *Staphylococcus*, and *Stenotrophomonas*. The sequences obtained from isolates and sample B of *T. variabilis* formed eight OTUs, two belonging to the genus *Bacillus* and one belonging to each of the following genus: *Aquitalea*, *Chromobacterium*, *Chryseobacterium*, *Enterobacter*, *Klebsiella*, *Stenotrophomonas*. Finally, five OTUs were shared between the sequences obtained from the isolates and the sponge sample C, they were identified as *Aquitalea*, *Chromobacterium*, *Dickeya*, *Enterobacter*, and *Klebsiella*. However, no OTU was unique to that sponge individual.

Despite that, it can be observed that some bacterial groups were common among the samples. For example, *Pseudomonas* (Gammaproteobacteria) was a genus observed only in the freshwater samples in both analyses (culture-dependent and independent). In addition, bacteria from the phyla Bacteroidetes and Firmicutes were also commonly isolated from both samples (sponges and surrounding freshwater). Analyzing the culture-independent data, the presence of the phylum Firmicutes was not very noteworthy among the main OTUs analyzed, although this phylum was the second most frequent in terms of relative abundance when isolated by culture.

### Antimicrobial Substances Produced by Isolates

All 163 strains identified were selected for antimicrobial activity assay against *S. aureus* (Supplementary Figure S4). Twenty-one (20.2%, 21/104) sponge-associated bacteria showed inhibitory activity, with strains mainly belonging to genera *Aquitalea, Chromobacterium*, *Dickeya*, *Klebsiella*, and to the family *Enterobacteriaceae*.

Inhibitory activities were also observed in 25.4% (15/59) of the strains isolated from freshwater and a largest number of bioactive isolates were affiliated with genera *Chryseobacterium*, *Enterobacter*, *Klebsiella*, *Pseudomonas*, and *Stenotrophomonas*.

The list of bioactive isolates and the measures of the diameter of inhibition zone of *S. aureus* growth are presented in Supplementary Table S2.

## Discussion

Previous studies with freshwater sponge-associated microorganisms were restricted to a small group of bacteria (Gernert et al., 2005; Costa et al., 2012; Keller-Costa et al., 2014; Kulakova et al., 2014; Gaikwad et al., 2016). In addition, it was believed that the bacterial diversity of freshwater sponges was lower than those of marine sponges (Taylor et al., 2007; Gaikwad et al., 2016). The present study provides a comparison of bacterial communities in three different *T. variabilis* samples, a freshwater sponge, and their surrounding waters. A high bacterial diversity was observed in the sponges. Moreover, there were clear differences between diversity and richness comparing *T. variabilis* and the surrounding water, supporting previous publications (Gladkikh et al., 2014; Gaikwad et al., 2016) that also analyzed the microbial diversity of other freshwater sponges using NGS technology.

In general, our results are in accordance with other studies on sponge-associated bacteria and surrounding water collected from different environments where Proteobacteria were highly dominant in terms of relative abundance (Sipkema et al., 2009; Cárdenas et al., 2014; Moitinho-Silva et al., 2014, 2017; Rodríguez-Marconi et al., 2015; Thomas et al., 2016; De Mares et al., 2017; Lurgi et al., 2019). Alphaproteobacteria was the dominant class in *T. variabilis* while Betaproteobacteria was dominant in freshwater. Marine sponges mostly harbored Alphaproteobacteria and Gammaproteobacteria (Thomas et al., 2016; Moitinho-Silva et al., 2017; Lurgi et al., 2019), while the freshwater environment was well documented to possess a wide distribution of Alphaproteobacteria and Betaproteobacteria due to its pH and nutrient contents (Newton et al., 2011). In minor abundance, Bacteroidetes, Acidobacteria, Verrucomicrobia, Cyanobacteria, and further 39 phyla and candidate phyla were also observed in this study. In this regard, based on the abundance of dominant groups, the phyla detected are phyla that have so far been detected in 81 different marine sponge species, as well as seawater and marine sediments (Wang et al., 2012; Thomas et al., 2016). In another study, significant differences between the microbial community structure of the two freshwater sponge species *E. carteri* and *C. lapidosa* were observed. *E. carteri* showed an abundance of Firmicutes followed by Proteobacteria and Cyanobacteria, while *C. lapidosa* showed a greater abundance of Proteobacteria followed by Planctomycetes, Cyanobacteria, and Actinobacteria (Gaikwad et al., 2016).

The structure of the bacterial community showed higher variation (beta-diversity) in *T. variabilis* samples compared to water samples. Variation in the microbial composition of the sponge suggests a more unrestricted relationship of the host with their microbiome, with a vertical transmission, which means that different bacteria can colonize the *T. variabilis*, and not only a very specific group (Thacker and Freeman, 2012). When the top thirty OTUs were compared among the sponge and freshwater samples, 20 showed significant differences between the two habitats and most of those were very abundant in one of them and almost absent in the other. A little overlap in microbiomes between sponges and the surrounding water was also noted for marine sponges (Thomas et al., 2016; Moitinho-Silva et al., 2017; Lurgi et al., 2019). The main OTUs enriched in *T. variabilis* belonged to genus *Methylosinus*, a methanotrophic bacteria that acquires copper from the surrounding habitat that is essential for its cells (Semrau et al., 2010). The second most enriched OTU belonged to family *Cytophagacea*, which is one of the largest families in the phylum Bacteroidetes and many of its members digest macromolecules such as polysaccharides or proteins (McBride et al., 2014). The main OTUs found to be enriched in freshwater belonged to genera *Methylocaldum*, *Sulfuricurvum*, C39 and family *Comamonadacea*. Bacteria of the genus *Methylocaldum* are obligate methanotrophs, using methane as their sole carbon and energy source (Hanson and Hanson, 1996). *Sulfuricurvum* is characterized as anaerobic/microaerophilic and sulfur-oxidizing chemolithoautotroph. This genus can use various electron receptors (e.g., oxygen, nitrate) and electron donors (e.g., elemental sulfur, sulfide, thiosulfate), and are widely spread in subsurface environments (Engel et al., 2003; Kodama and Watanabe, 2004; Krauze et al., 2017).

The richness and structure of the microbial community of *T. variabilis* were also compared to that of two other freshwater sponges (*E. carteri* and *C. lapidosa*) and 32 different marine sponges to understand how they are related. We identified a higher microbial richness in *T. variabilis* samples when compared to other sponge species. Number of OTUs obtained were higher than reported in other studies on marine sponges collected from different oceans (Thomas et al., 2016). These results are in agreement with meta-analysis data, which showed that bacterial communities in freshwater have higher levels of diversity than those of saline waters (Barberan and Casamayor, 2010). As a consequence of continued colonization by microbiota over millions of years, the aspects that contribute to the large microbial diversity in freshwater sponges include characteristics of the sponge, of the associated microbial community and of the continuous exposure of the sponge to its surrounding water (Hentschel et al., 2012; Gaikwad et al., 2016). A study recently showed that abiotic factors affect the structure of the microbial community of marine sponges. Nevertheless, biotic interactions are the ones that control the composition of closely associated “core” microorganisms. These findings imply that both ecological and evolutionary processes are at play in sponge-associated bacteria (Lurgi et al., 2019).

It is known that the freshwater sediments harbor greater microbial diversity compared to intertidal and marine sediments. Thus, there will be a remote possibility that this environment external to the sponge could serve as a source of microorganisms for the colonization of the microbiota of freshwater sponges (Wang et al., 2012; Gaikwad et al., 2016). This proposal would also be related to a greater microbial diversity found in freshwater sponges when compared to that in marine sponges (Gaikwad et al., 2016). Here, we suggest that the microbial community of the *T. variabilis* sponge is influenced by both vertical and horizontal transfer of the microorganisms. However, there are no studies on freshwater sponge-microbe transmission to demonstrate such proposals. Thacker and Freeman (2012) called it leaky vertical transmission. This type of sponge-microbe transmission is predominantly vertical with an occasional environmental acquisition, or vertical with massive environmental swamping. Host species that exhibit various degrees of leaky vertical transmission might reflect diverse evolutionary solutions that balance the costs and benefits of purely vertical versus horizontal modes of transmission (Vrijenhoek, 2010; Thacker and Freeman, 2012).

Another hallmark of the present work on the global microbiome structure analysis was that the microbial community of the sponges was clustered in two different groups, one with the three freshwater sponges (*E. carteri*, *C. lapidosa*, and *T. variabilis*) and other with the marine sponges. Based on the fact that *T. variabilis* (collected in Brazil) is geographically very distant from the other two freshwater sponge species (collected in India), we demonstrate that a possible factor structuring of the microbial community is the type of aquatic ecosystems, maybe the water salinity. This cluster of two distinct groups is unprecedented in the literature and should be further investigated as more studies on the microbiome of freshwater sponges are performed.

To date, it is well established that only approximately 1% of the bacteria on Earth can be readily cultivated *in vitro*. Current estimates point to 92 bacterial phyla, of which at least half have no cultivable representatives (Vartoukian et al., 2010; Hug et al., 2016). However, many publications have shown that a high percentage of members of the host-associated microbiota may be isolated in artificial culture media and these culture collections are crucial for eliciting important features of microbiome function (Taylor et al., 2007; Webster and Taylor, 2012; Montalvo et al., 2014; Graça et al., 2015; Laport et al., 2016; Laport, 2017; Carini, 2019).

In the present work, the use of five different culture media contributed to the isolation of a high bacterial diversity as previously described in other studies (Montalvo et al., 2014; Graça et al., 2015; Laport et al., 2016; Laport, 2017). Twenty-three genera were recovered from *T. variabilis* and 14 from freshwater samples, with 12 genera being exclusively isolated from sponges and three exclusively from freshwater. Bacteria commonly isolated from sponges typically belong to the Actinobacteria, Bacteroidetes, Firmicutes or Proteobacteria (Taylor et al., 2007; Webster and Taylor, 2012; Montalvo et al., 2014; Laport, 2017). In our study, Proteobacteria (Alpha-, Beta-, Delta-, Gammaproteobacteria classes) was also the main phylum isolated and identified among the strains, as observed in the NGS analysis. However, the relative abundances of the Proteobacteria classes detected by the two methodologies did not follow the same pattern. In the NGS analysis there was a predominance of Alphaproteobacteria and Betaproteobacteria, whereas the bacteria recovered from culture media showed a greater abundance of Gammaproteobacteria followed by Betaproteobacteria. Although only three OTUs were shared among all cultured and non-cultured bacteria, all belonged to Proteobacteria. They were identified as belonging to the genera *Aquitelea* (Betaproteobacteria), *Chromobacterium* (Betaproteobacteria), and *Enterobacter* (Gammaproteobacteria). In contrast, another study on the biodiversity of the marine sponge microbial community, also by integration of culture-based and molecular analysis, observed that Gammaproteobacteria was the only group of cultured isolates represented in the 454 sequences (Montalvo et al., 2014). Perhaps, these data may reflect the good bacterial recovery obtained by the culture-dependent methodology applied in the present study. Another result found that what strengthens our culture-dependent analysis was the fact that *Pseudomonas* (Gammaproteobacteria) was a genus observed only in the freshwater samples in both analysis (culture-dependent and independent).

In the culture-dependent analysis with sponges and freshwater samples, the most frequently isolated bacteria were affiliated predominately with the *Enterobacteriaceae* family. Some members of this bacterial family of the class Gammaproteobacteria are indigenous gut microbes in animals and humans, and other members are also found in water or soil, and can be parasites of various animals and plants (Kittinger et al., 2016). The aquatic environment is an *Enterobacteriaceae* reservoir, a fact that has been overlooked in past times. Our results are important for the reflection about the role of this bacterial family as an environmental bioindicator of pollution. It is known that the water of the da Prata River basin that empties in the artificial channel that provides water for the fish farm of Universidade Federal Rural de Pernambuco showed some changes in its quality, because of anthropogenic actions (SOS Mata Atlântica, 2012).

In addition, bacteria from the phyla Bacteroidetes and Firmicutes were also commonly isolated in both samples (sponges and freshwater) and few OTUs were shared between the cultured and non-cultured communities. Analyzing the NGS data, the phylum Firmicutes were not representative among the main OTUs analyzed, whereas they were the second most frequently isolated in terms of relative abundance, probably because of the composition of the culture media used in this study that favored the isolation of this group. Another prominent phylum among the isolated bacteria from *T. variabilis* was the Actinobacteria, although just one OTU was shared between culture-dependent and independent analysis. Aquatic Actinobacteria are recognized as a treasure house of secondary metabolites due to their capability to produce novel bioactive molecules, notably antibiotics, antitumor agents, immunosuppressive agents and enzymes (Laport, 2017).

It is more surprising that the culture-based work undertaken here resulted in the isolation of groups of bacteria that are not detected by even the deep sequencing community analysis. There are two possible explanations for this, according to Montalvo et al. (2014). First, the cultured bacteria may be present itself as very minor constituents of the sponge-associated bacterial community. Many of these bacterial groups were repeatedly isolated from different individuals of the same sponge species. Second, pyrosequencing analysis may result in significant biases leading to the lack of detection of entire bacterial groups. This suggests that they are consistently associated with the sponges and may play an important role in the bacterial community, even if they are present at very low numbers (Montalvo et al., 2014). The physicochemical conditions found in the aquatic environment, such as variations in temperature, salinity, pH, pressure, luminosity, nutrient availability and space competition, are important factors that lead to an improvement in strategies by bacteria in order to colonize and grow in its habitat. In addition, bacteria that produce bioactive substances are capable of inhibiting the growth of other bacteria present in the same environment, and this competitive success may confer an important ecological advantage (Laport et al., 2016). Very few studies have addressed the isolation of freshwater bacteria and the analysis of the production of antimicrobial substances. In the present work, inhibitory activity in a range of 20–25% was detected among bacteria isolated from sponges and freshwater. Bacteria presenting antimicrobial activity belonged to five different genera among which the genus *Klebsiella* predominated. It has already been proposed that the sharing of bacterial genera with antimicrobial activity among sponges and the surrounding water should play some ecological role for that particular microbial community (Laport et al., 2016). Some of the genera are known to produce bioactive substances, such as anti-quorum-sensing compounds from *Chromobacterium* (El-Gohary and Shaaban, 2018) and antibacterial compounds from *Enterobacter* (Laport et al., 2017) and *Pseudomonas* (Marinho et al., 2009; Graça et al., 2015; Santos et al., 2015), including species isolated from *Ephydatia fluviatilis*, a freshwater sponge (Keller-Costa et al., 2014). In this study, the inhibitory activity from aquatic bacteria was observed against *S. aureus*, an important pathogenic bacterial species commonly associated with antimicrobial resistance. Research and development are needed to produce and characterize new compounds that can be implemented against multidrug resistant bacteria (World Health Organization [WHO], 2017). These data open up interesting avenues in the search for novel antimicrobials.

Deeper knowledge about the ecology and genetic diversity of these microorganisms, their role in the health of the host sponge and the production of natural substances, requires laboratory cultivation. The culture provides access to genetic and biochemical characteristics of each microorganism that may not be revealed by the 16S rRNA gene massive sequencing. In addition to the production of bioactive substances, the isolates associated with the cultured sponge also revealed ecological relevance (Taylor et al., 2007; Montalvo et al., 2014; Laport, 2017; Carini, 2019).

This present work not merely demonstrates that the microbial composition of *T. variabilis* is highly diverse and different from that found in the surrounding freshwater, but also suggests that the type of aquatic ecosystems, as water salinity, can be a limiting factor in the differentiation of the bacterial community structure between freshwater sponges and marine sponges. This study also highlights the importance of culture-dependent analysis for isolation of representatives of the novel bacterial groups found in freshwater sponges. As previously noted, culturing is adding new insights about sponge bacteria symbiont relationship and the research of new bioactive substances, especially in this group of sponges with scarce information on their microbiota. Moreover, the inhibitory activity observed for the bacterial strains might play an important role in the shaping microbial communities of that environment. Further efforts to study representatives of the key groups of bacteria found in freshwater sponges are necessary.

## Data Availability Statement

The datasets generated for this study can be found in the NCBI Sequence Read Archive (SRA): SRP115997, NCBI database GenBank: MG099655, MG099656, MG099657, MH424474-MH424486, MH426850-MH426928, MH454615-MH454640, MH470383-MH470403, and MH477670-MH477693.

## Ethics Statement

The collection site is public and there is no requirement for a Porifera collection, however, one of the authors, UP has a permanent license for the collection of zoological material under the number 18100-1, issued by the System of Authorization and Information on Biodiversity (*Sistema de Autorização e Informação em Biodiversidade* – SISBIO) of the Ministry of Environment (*Ministério do Meio Ambiente* – MMA), Brazil.

## Author Contributions

ML, UP, and CR: study conception, design, and critical revision. ML and UP: acquisition and identification of sponge samples. ML: provided reagents and materials. ML and CR: acquisition of data (experimental development), analyses and interpretation of data, and drafting of the manuscript.

## Conflict of Interest

The authors declare that the research was conducted in the absence of any commercial or financial relationships that could be construed as a potential conflict of interest.

## Footnotes

1 http://www.arb-silva.de/aligner/

## Supplementary Material

FIGURE S1 Richness and Diversity indexes of the microbiome of *Tubella variabilis* and surrounding freshwater. **(a)** Richness measured by number of OTU. **(b)** Diversity measured by Shannon index.

FIGURE S2 Rarefaction curves of 16S rRNA sequences. The number of different OTUs is given as a function of the number of sequences obtained by Illumina sequencing. Each colored line represents the OTUs from *T. variabilis* (blue) and surrounding freshwater (orange).

FIGURE S3 Non-metric multidimensional scaling based on OTU distribution among freshwater and *T. variabilis* samples. Stress value is given in 0–1 scale.

FIGURE S4 Representative images of the antimicrobial activity assay with bacteria isolated from *T. variabilis* and surrounding water samples against *Staphylococcus aureus* ATCC29213. The indicator strain *S. aureus* was considered sensitive to the activity of the producer strain when it exhibited a clear inhibition zone around the spotted strain with a diameter ≥8 mm.

TABLE S1 List of sponge hosts used in the global analysis comparing freshwater and marine sponges, with the access number and the databased used to retrieve the sequences.

TABLE S2 Antimicrobial activity assay performed with bacteria isolated from sponges and freshwater against *Staphylococcus aureus* ATCC29213.

## References

[B1] Barberan A. Casamayor E. O. (2010). Global phylogenetic community structure and beta-diversity patterns in surface bacterioplankton metacommunities. *Aquat Microb. Ecol.* 59 1–10. 10.3354/ame01389

[B2] Bonetto A. A. Ezcurra de Drago I. D. (1973). Las esponjas del género *Trochospongilla* vejdovsky en águas argentinas. *Physis* 32 13–18.

[B3] Caporaso J. G. Lauber C. L. Walters W. A. Berg-Lyons D. Lozupone C. A. Turnbaugh P. J. (2011). Global patterns of 16S rRNA diversity at a depth of millions of sequences per sample. *Proc. Natl. Acad. Sci. U.S.A.* 108 4516–4522. 10.1038/ismej.2012.8 20534432 PMC3063599

[B4] Cárdenas C. A. Bell J. J. Davy S. K. Hoggard M. Taylor M. W. (2014). Influence of environmental variation on symbiotic bacterial communities of two temperate sponges. *FEMS Microbiol. Ecol.* 88 516–527. 10.1111/1574-6941.12317 24617641

[B5] Carini P. (2019). A “cultural” renaissance: genomics breathes new life into an old craft. *mSystems* 4:e00092-19. 10.1128/mSystems.00092-19 31219785 PMC6533372

[B6] Cole J. R. Wang Q. Cardenas E. Fish J. Chai B. Farris R. J. (2009). The ribosomal database project: improved alignments and new tools for rRNA analysis. *Nucleic Acids Res.* 37 D141–D145. 10.1093/nar/gkn879 19004872 PMC2686447

[B7] Costa R. Keller-Costa T. van Overbeek L. van Elsas J. D. (2012). Evidence for selective bacterial community structuring in the freshwater sponge *Ephydatia fluviatilis*. *Microb. Ecol.* 65 232–244. 10.1007/s00248-012-0102 22903086

[B8] De Mares C. M. Sipkema D. Huang S. Bunk B. Overmann J. van Elsas J. D. (2017). Host specificity for bacterial, archaeal and fungal communities determined for high- and low-microbial abundance sponge species in two genera. *Front. Microbiol.* 8:2560. 10.3389/fmicb.2017.02560 29326681 PMC5742488

[B9] El-Gohary N. S. Shaaban M. I. (2018). New pyrazolopyridine analogs: synthesis, antimicrobial, antiquorum-sensing and antitumor screening. *Eur. J. Med. Chem.* 152 126–136. 10.1016/j.ejmech.2018.04.025 29702448

[B10] Engel A. S. Lee N. Porter M. L. Stern L. A. Bennett P. C. Wagner M. (2003). Filamentous “*Epsilonproteobacteria*” dominate microbial mats from sulfidic cave springs. *Appl. Environ. Microbiol.* 69 5503–5511. 10.1128/AEM.69.9.5503-5511.2003 12957939 PMC194925

[B11] Folmer O. Black M. Hoeh W. Lutz R. Vrijenhoek R. (1994). DNA primers for amplification of mitochondrial cytochrome c oxidase subunit I from diverse metazoan invertebrates. *Mol. Mar. Biol. Biotechnol.* 3 294–299. 7881515

[B12] Fukami H. Budd A. F. Levitan D. R. Jara J. Kersanach R. Knowlton N. (2004). Geographic differences in species boundaries among members of the *Montastraea annularis* complex based on molecular and morphological markers. *Evolution* 58 324–337. 10.1554/03-026 15068349

[B13] Gaikwad S. Shouche Y. S. Gade W. N. (2016). Microbial community structure of two freshwater sponges using Illumina MiSeq sequencing revealed high microbial diversity. *AMB Express* 6:40. 10.1186/s13568-016-0211-2 27299740 PMC4908081

[B14] Gazave E. Lapébie P. Ereskovsky A. V. Vacelet J. Renard E. Cárdenas P. (2012). No longer demospongiae: homoscleromorpha formal nomination as a fourth class of porifera. *Hydrobiologia* 687 3–10. 10.1007/s10750-011-0842-x

[B15] Gernert C. Glockner F. O. Krohne G. Hentschel U. (2005). Microbial diversity of the freshwater sponge *Spongilla lacustris*. *Microb. Ecol.* 50 206–212. 10.1007/s00248-004-0172-x 16211324

[B16] Gladkikh A. S. Kalyuzhnaya O. V. Belykh O. I. Ahn T. S. Parfenova V. V. (2014). Analysis of bacterial communities of two lake Baikal endemic sponge species. *Mikrobiologiia.* 83 787–797. 10.1134/s002626171406006x 25941718

[B17] Graça A. P. Viana F. Bondoso J. Correia M. I. Gomes L. Humanes M. (2015). The antimicrobial activity of heterotrophic bacteria isolated from the marine sponge Erylus deficiens (*Astrophorida*, *Geodiidae*). *Front. Microbiol.* 7:e389 10.3389/fmicb.2015.00389 PMC4423441 25999928

[B18] Hammer Ø Harper D. A. T. Ryan P. D. (2001). PAST: paleontological statistics software package for education and data analysis. *Palaeontol. Electron.* 4 1–9.

[B19] Hanson R. S. Hanson T. E. (1996). Methanotrophic bacteria. *Microbiol. Rev.* 60 439–471. 8801441 10.1128/mr.60.2.439-471.1996 PMC239451

[B20] Hentschel U. Piel J. Degnan S. M. Taylor M. W. (2012). Genomic insights into the marine sponge microbiome. *Nat. Rev. Microbiol.* 10 641–654. 10.1038/nrmicro2839 22842661

[B21] Hug L. A. Baker B. J. Anantharaman K. Brown C. T. Probst A. J. Castelle C. J. (2016). A new view of the tree of life. *Nat. Microbiol.* 1:16048. 10.1038/nmicrobiol.2016.48 27572647

[B22] Kaluzhnaya O. V. Krivich A. A. Itskovich V. B. (2012). Diversity of 16S rRNA genes in metagenomic community of the freshwater sponge *Lubomirskia baicalensis*. *Genetika* 48 1003–1006. 23035553

[B23] Keller-Costa T. Jousset A. van Overbeek L. van Elsas J. D. Costa R. (2014). The freshwater sponge *Ephydatia fluviatilis* harbours diverse *Pseudomonas* species (*Gammaproteobacteria*, *Pseudomonadales*) with broad-spectrum antimicrobial activity. *PLoS One* 9:e88429. 10.1371/journal.pone.0088429 24533086 PMC3922812

[B24] Kittinger C. Lipp M. Folli B. Kirschner A. Baumert R. Galler H. (2016). Enterobacteriaceae isolated from the river Danube: antibiotic resistances, with a focus on the presence of ESBL and carbapenemases. *PLoS One* 11:e0165820. 10.1371/journal.pone.0165820 27812159 PMC5094594

[B25] Kodama Y. Watanabe K. (2004). *Sulfuricurvum kujiense* gen. nov., sp. nov., a facultatively anaerobic, chemolithoautotrophic, sulfur-oxidizing bacterium isolated from an underground crude-oil storage cavity. *Int. J. Syst. Evol. Microbiol.* 54 2297–2300. 10.1099/ijs.0.63243-0 15545474

[B26] Krauze P. Kämpf H. Horn F. Liu Q. Voropaev A. Wagner D. (2017). Microbiological and geochemical survey of CO2-Dominated mofette and mineral waters of the Cheb Basin, Czech Republic. *Front. Microbiol.* 8:2446. 10.3389/fmicb.2017.02446 29321765 PMC5732176

[B27] Kulakova N. V. Denikina N. N. Belikov S. I. (2014). Diversity of bacterial photosymbionts in lubomirskiidae sponges from lake Baikal. *Int. J. Biodiv.* 2014:152097 10.1155/2014/152097

[B28] Laport M. S. (2017). Isolating bacteria from sponges: why and how? *Curr. Pharm. Biotechnol.* 18 1224–1236. 10.2174/1389201019666180329111327 29595106

[B29] Laport M. S. Bauwens M. de Oliveira N. S. Willenz P. George I. Muricy G. (2017). Culturable bacterial communities associated to Brazilian *Oscarella* species (*Porifera*: *Homoscleromorpha*) and their antagonistic interactions. *Antonie Van Leeuwenhoek* 110 489–499. 10.1007/s10482-016-0818-y 28008548

[B30] Laport M. S. Santos-Gandelman J. F. Muricy G. Giambiade-deMarval M. George I. (2016). Antagonistic interactions among bacteria isolated from either the same or from different sponges native to the Brazilian coast. *J. Mar. Sci. Res. Dev.* 6:185 10.4172/2155-9910.1000185

[B31] Lurgi M. Thomas T. Wemheuer B. Webster N. S. Montoya J. M. (2019). Modularity and predicted functions of the global sponge-microbiome network. *Nat. Commun.* 10:992. 10.1038/s41467-019-08925-4 30824706 PMC6397258

[B32] Manconi R. Pronzato R. (2008). Global diversity of sponges (*Porifera*: *Spongillina*) in freshwater. *Hydrobiologia.* 595 27–33. 10.1007/978-1-4020-8259-7_3

[B33] Marinho P. R. Moreira A. P. Pellegrino F. L. Muricy G. Bastos M. C. Santos K. R. (2009). Marine *Pseudomonas putida*: a potential source of antimicrobial substances against antibiotic-resistant bacteria. *Mem. Inst. Oswaldo Cruz.* 104 678–682. 10.1590/s0074-02762009000500002 19820824

[B34] McBride M. J. Liu W. Lu X. Zhu Y. Zhang W. (2014). “The family cytophagaceae,” in *The Prokaryotes – Other Major Lineages of Bacteria and the Archaea*, eds Rosenberg E. DeLong E. F. Lory S. Stackebrandt E. Thompson F. (Dordrecht: Springer), 577–593. 10.1007/978-3-642-38954-2_382

[B35] Moitinho-Silva L. Nielsen S. Amir A. Gonzalez A. Ackermann G. L. Cerrano C. (2017). The sponge microbiome project. *Gigascience* 6 1–7. 10.1093/gigascience/gix077 29020741 PMC5632291

[B36] Moitinho-Silva L. Seridi L. Ryu T. Voolstra C. R. Ravasi T. Hentschel U. (2014). Revealing microbial functional activities in the Red Sea sponge *Stylissa carteri* by metatranscriptomics. *Environ. Microbiol.* 6 3683–3698. 10.1111/1462-2920.12533 24920529

[B37] Montalvo N. F. Davis J. Vicente J. Pittiglio R. Ravel J. Hill R. T. (2014). Integration of culture-based and molecular analysis of a complex sponge-associated bacterial community. *PLoS One* 9:e90517. 10.1371/journal.pone.0090517 24618773 PMC3949686

[B38] Newton R. J. Jones S. E. Eiler A. McMahon K. D. Bertilsson S. (2011). A guide to the natural history of freshwater lake bacteria. *Microbiol. Mol. Biol. Rev.* 75 14–49. 10.1128/MMBR.00028-10 21372319 PMC3063352

[B39] Nicacio G. Pinheiro U. (2015). Biodiversity of freshwater sponges (porifera: *Spongillina*) from northeast Brazil: new species and notes on systematics. *Zootaxa* 3981 220–240. 10.11646/zootaxa.3981.2.4 26249990

[B40] Pruesse E. Quast C. Knittel K. Fuchs B. M. Ludwig W. Peplies J. (2007). SILVA: a comprehensive online resource for quality checked and aligned ribosomal RNA sequence data compatible with ARB. *Nucleic Acids Res.* 2007 7188–7196. 10.1093/nar/gkm864 17947321 PMC2175337

[B41] Quast C. Pruesse E. Yilmaz P. Gerken J. Schweer T. Yarza P. (2013). The SILVA ribosomal RNA gene database project: improved data processing and web-based tools. *Nucleic Acids Res.* 2013 D590–D596. 10.1093/nar/gks1219 23193283 PMC3531112

[B42] Rodríguez-Marconi S. De la Iglesia R. Díez B. Fonseca C. A. Hajdu E. Trefault N. (2015). Characterization of bacterial, archaeal and eukaryote symbionts from Antarctic sponges reveals a high diversity at a three-domain level and a particular signature for this ecosystem. *PLoS One* 10:e0138837. 10.1371/journal.pone.0138837 26421612 PMC4589366

[B43] Santos O. C. S. Soares A. R. Machado F. L. S. Romanos M. T. V. Muricy G. Giambiagi-deMarval M. (2015). Investigation of biotechnological potential of metabolites extracted from sponge-associated bacteria collected in Brazilian coast. *Lett. Appl. Microbiol.* 60 140–147. 10.1111/lam.12347 25355062

[B44] Santos-Gandelman J. F. Giambiagi-deMarval M. Oelemann W. M. R. Laport M. S. (2014). Biotechnological potential of sponge-associated bacteria. *Curr. Pharmac. Biotechnol.* 15 143–155. 10.2174/1389201019666180329111327 25022270

[B45] Schloss P. D. Westcott S. L. Ryabin T. Hall J. R. Hartmann M. Hollister E. B. (2009). Introducing mothur: open-source, platform-independent, community-supported software for describing and comparing microbial communities. *Appl. Environ. Microbiol.* 75 7537–7541. 10.1128/AEM.01541-9 19801464 PMC2786419

[B46] Semrau J. D. DiSpirito A. A. Yoon S. (2010). Methanotrophs and copper. *FEMS Microbiol. Rev.* 34 496–531. 10.1111/j.1574-6976.2010.00212.x 20236329

[B47] Shannon C. E. (1948). A mathematical theory of communication. *Bell. Syst. Tech. J.* 27 379–423.

[B48] Sipkema D. Holmes B. Nichols S. A. Blanch H. W. (2009). Biological characterisation of haliclona (?gellius) sp.: sponge and associated microorganisms. *Microb. Ecol.* 58 903–920. 10.1007/s00248-009-9534-8 19471996 PMC2772955

[B49] SOS Mata Atlântica (2012). *Resultado Da Análise Da Água Do Açude Do Prata, Recife-PE.* Available at: https://www.sosma.org.br/blog/resultado-da-analise-da-agua-do-acude-do-prata-recife-pe/ (accessed February 28, 2019).

[B50] Taylor M. W. Radax R. Steger D. Wagner M. (2007). Sponge associated microorganisms: evolution, ecology, and biotechnological potential. *Microbiol. Mol. Biol. Rev.* 71 295–347. 10.1128/mmbr.00040-06 17554047 PMC1899876

[B51] Thacker R. W. Freeman C. J. (2012). Sponge-microbe symbioses. recent advances and new directions. *Adv. Mar. Biol.* 62 57–110. 10.1016/B978-0-12-394283-8.00002-3 22664121

[B52] Thomas T. Moitinho-Silva L. Lurgi M. Björk J. R. Easson C. Astudillo-García C. (2016). Diversity, structure and convergent evolution of the global sponge microbiome. *Nat. Commun.* 7:11870. 10.1038/ncomms11870 27306690 PMC4912640

[B53] Vacelet J. (1975). Étude en microscopie électronique de l’association entre bactéries et spongiaires du genre *Verongia* (*Dictyoceratida*). *J. Microsc. Biol. Cell.* 23 271–288.

[B54] van Soest R. W. M. Boury-Esnault N. Hooper J. N. A. Rützler K. de Voogd N. J. Alvarez B. (2019). *World Porifera Database.* Available at: http://www.marinespecies.org/porifera. (accessed July 16, 2019).

[B55] van Soest R. W. M. Boury-Esnault N. Vacelet J. Dohrmann M. Erpenbeck D. de Voogd N. J. (2012). Global diversity of sponges (*Porifera*). *PLoS One* 7:e35105. 10.1371/journal.pone.0035105 22558119 PMC3338747

[B56] Vartoukian S. R. Palmer R. M. Wade W. G. (2010). Strategies for culture of “unculturable” bacteria. *FEMS Microbiol. Lett.* 309 1–7. 10.1111/j.1574-6968.2010.02000.x 20487025

[B57] Vrijenhoek R. C. (2010). “The vent and seep biota,” in *Aspects from Microbes to Ecosystems*, ed. Kiel S. (Dordrecht: Springer), 15–49.

[B58] Wang Y. Sheng H. He Y. Wu J. Y. Jiang Y. X. Tam N. F. (2012). Comparison of the levels of bacterial diversity in freshwater intertidal wetland and marine sediments by using millions of Illumina tags. *Appl. Environ. Microbiol.* 78 8264–8271. 10.1128/AEM.01821-12 23001654 PMC3497375

[B59] Webster N. S. Taylor M. W. (2012). Marine sponges and their microbial symbionts: love and other relationships. *Environ. Microbiol.* 14 335–346. 10.1111/j.1462-2920.2011.02460.x 21443739

[B60] Weisburg W. G. Barns S. M. Pelletier D. A. Lane D. J. (1991). 16S ribosomal DNA amplification for phylogenetic study. *J. Bacteriol.* 173 697–703. 10.1128/jb.173.2.697-703.1991 1987160 PMC207061

[B61] World Health Organization [WHO], (2017). *Prioritization of Pathogens to Guide Discovery, Research and Development of New Antibiotics for Drug-Resistant Bacterial Infections, Including Tuberculosis.* (Geneva: World Health Organization).

[B62] Wilkinson C. R. Garrone R. Vacelet J. (1984). Marine sponges discriminate between food bacteria and bacterial symbionts: electron microscope radioautography and in situ evidence. *Proc. R. Soc. Lond. Ser. B Biol. Sci.* 220 519–528. 10.1098/rspb.1984.0018

