# Supplementary figures and images for: Freshwater Sponge Tubella variabilis Presents Richer Microbiota Than Marine Sponge Species
Source: Front Microbiol. 2019 Dec 3;10:2799. doi: 10.3389/fmicb.2019.02799 (PMC6902092; doi:10.3389/fmicb.2019.02799)

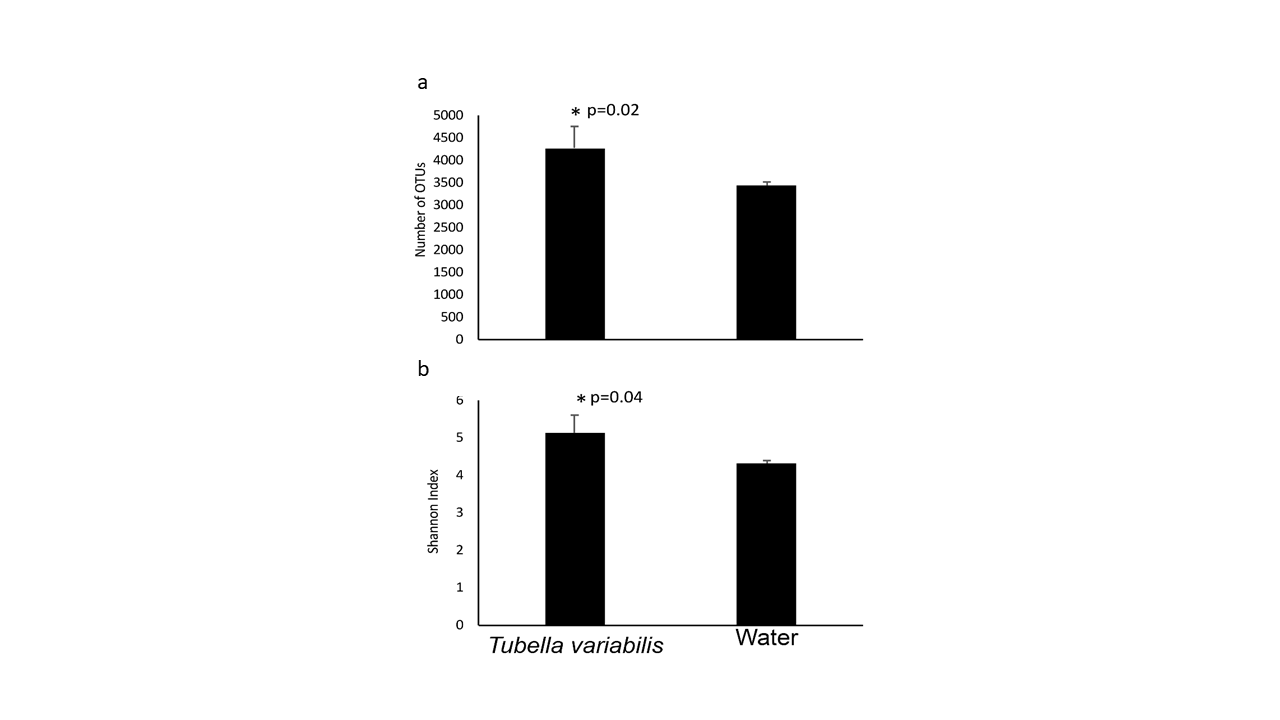

Supplement: FIGURE S1 — Richness and Diversity indexes of the microbiome of Tubella variabilis and surrounding freshwater. (a) Richness measured by number of OTU. (b) Diversity measured by Shannon index. [file Image_1.TIF]

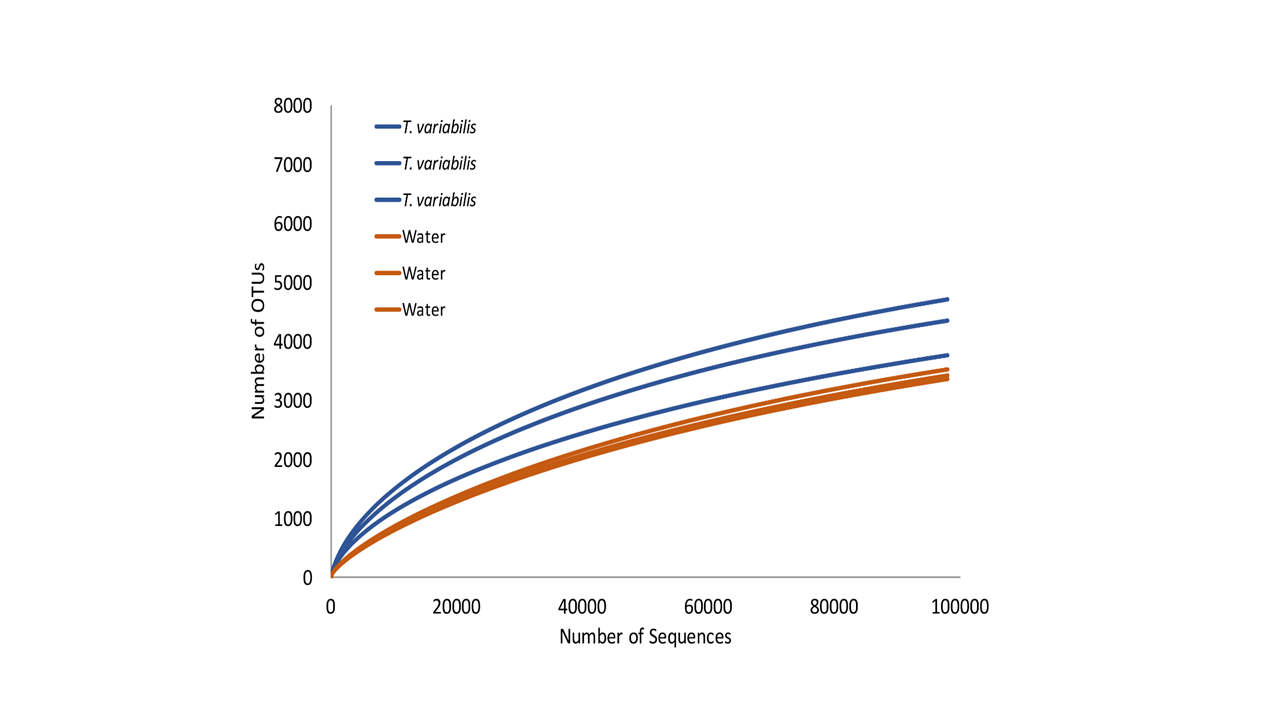

Supplement: FIGURE S2 — Rarefaction curves of 16S rRNA sequences. The number of different OTUs is given as a function of the number of sequences obtained by Illumina sequencing. Each colored line represents the OTUs from T. variabilis (blue) and surrounding freshwater (orange). [file Image_2.TIF]

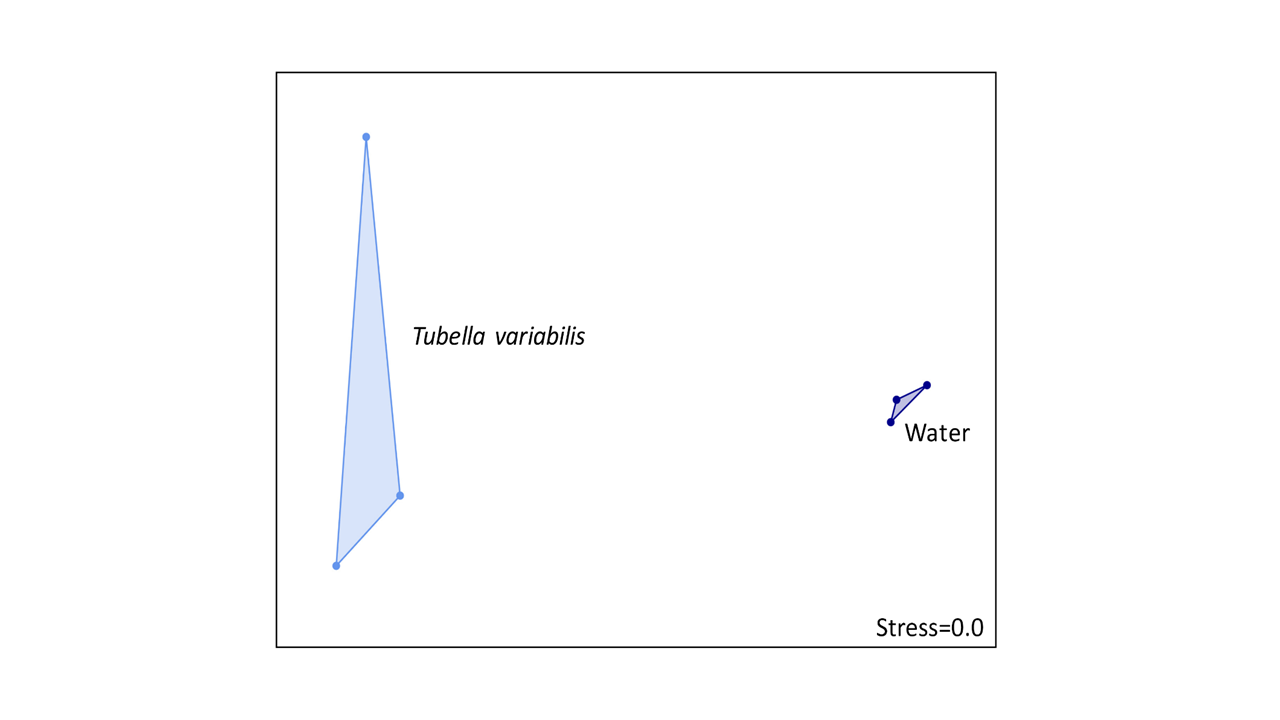

Supplement: FIGURE S3 — Non-metric multidimensional scaling based on OTU distribution among freshwater and T. variabilis samples. Stress value is given in 0–1 scale. [file Image_3.TIF]

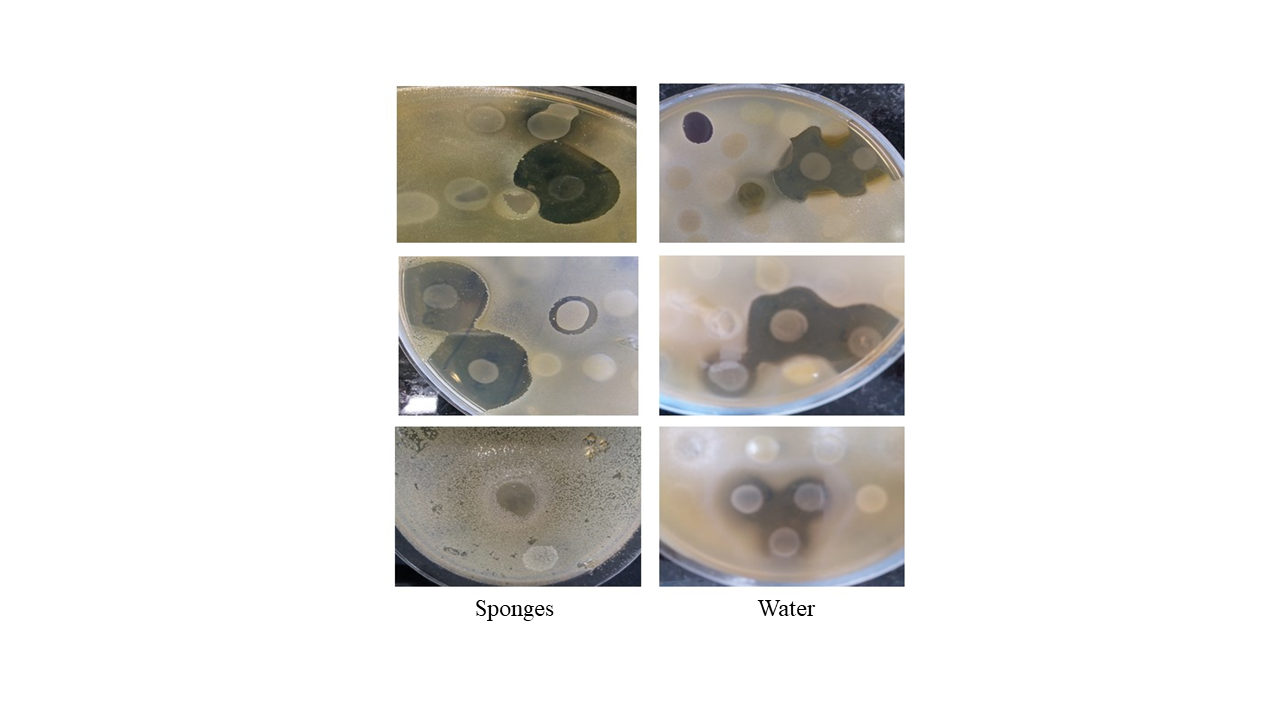

Supplement: FIGURE S4 — Representative images of the antimicrobial activity assay with bacteria isolated from T. variabilis and surrounding water samples against Staphylococcus aureus ATCC29213. The indicator strain S. aureus was considered sensitive to the activity of the producer strain when it exhibited a clear inhibition zone around the spotted strain with a diameter ≥8 mm. [file Image_4.TIF]
