# Supplementary material for: Freshwater Sponge Tubella variabilis Presents Richer Microbiota Than Marine Sponge Species
Source: Front Microbiol. 2019 Dec 3;10:2799. doi: 10.3389/fmicb.2019.02799 (PMC6902092; doi:10.3389/fmicb.2019.02799)
Supplement: TABLE S1 — List of sponge hosts used in the global analysis comparing freshwater and marine sponges, with the access number and the databased used to retrieve the sequences. [file Table_1.docx]

Table S1. List of sponge hosts used in the global analysis comparing freshwater and marine sponges, with the access number and the databased used to retrieve the sequences.

| Genus | Access Number | Database | Reference | Environment |
| --- | --- | --- | --- | --- |
| *Amphimedon* | ERR1159424 | NCBI / SRA | [2] | Marine |
| *Amphimedon* | ERR1159425 | NCBI / SRA | [2] | Marine |
| *Amphimedon* | ERR1159426 | NCBI / SRA | [2] | Marine |
| *Aplysina* | ERR1159336 | NCBI / SRA | [2] | Marine |
| *Aplysina* | ERR1159599 | NCBI / SRA | [2] | Marine |
| *Aplysina* | ERR1159337 | NCBI / SRA | [2] | Marine |
| *Axinella* | ERR1158954 | NCBI / SRA | [2] | Marine |
| *Axinella* | ERR1159644 | NCBI / SRA | [2] | Marine |
| *Axinella* | ERR1159646 | NCBI / SRA | [2] | Marine |
| *Biemna* | ERR1159618 | NCBI / SRA | [2] | Marine |
| *Carteriospongia* | ERR1160028 | NCBI / SRA | [2] | Marine |
| *Carteriospongia* | ERR1160029 | NCBI / SRA | [2] | Marine |
| *Carteriospongia* | ERR1160032 | NCBI / SRA | [2] | Marine |
| *Chondrilla* | ERR1159576 | NCBI / SRA | [2] | Marine |
| *Chondrilla* | ERR1159577 | NCBI / SRA | [2] | Marine |
| *Chondrilla* | ERR1159578 | NCBI / SRA | [2] | Marine |
| *Cinachyra* | ERR1160202 | NCBI / SRA | [2] | Marine |
| *Cinachyra* | ERR1160211 | NCBI / SRA | [2] | Marine |
| *Cinachyra* | ERR1160212 | NCBI / SRA | [2] | Marine |
| *Cliona* | ERR1159143 | NCBI / SRA | [2] | Marine |
| *Cliona* | ERR1159145 | NCBI / SRA | [2] | Marine |
| *Cliona* | ERR1159158 | NCBI / SRA | [2] | Marine |
| *Coelocarteria* | ERR1160201 | NCBI / SRA | [2] | Marine |
| *Coelocarteria* | ERR1160217 | NCBI / SRA | [2] | Marine |
| *Coelocarteria* | ERR1160218 | NCBI / SRA | [2] | Marine |
| *Corvospongilla* | [mgm4679572.3](https://www.mg-rast.org/mgmain.html?mgpage=overview&metagenome=mgm4679572.3) | MG-RAST | [1] | Freshwater |
| *Corvospongilla* | [mgm4679564.3](https://www.mg-rast.org/mgmain.html?mgpage=overview&metagenome=mgm4679564.3) | MG-RAST | [1] | Freshwater |
| *Corvospongilla* | [mgm4679566.3](https://www.mg-rast.org/mgmain.html?mgpage=overview&metagenome=mgm4679566.3) | MG-RAST | [1] | Freshwater |
| *Crambe* | ERR1159265 | NCBI / SRA | [17] | Marine |
| *Crambe* | ERR1159267 | NCBI / SRA | [2] | Marine |
| *Crambe* | ERR1159270 | NCBI / SRA | [2] | Marine |
| *Cymbastella* | ERR1159880 | NCBI / SRA | [2] | Marine |
| *Cymbastella* | ERR1159881 | NCBI / SRA | [2] | Marine |
| *Cymbastella* | ERR1159890 | NCBI / SRA | [2] | Marine |
| *Dysidea* | ERR1159149 | NCBI / SRA | [2] | Marine |
| *Dysidea* | ERR1159151 | NCBI / SRA | [2] | Marine |
| *Dysidea* | ERR1159341 | NCBI / SRA | [2] | Marine |
| *Erylus* | ERR1159472 | NCBI / SRA | [2] | Marine |
| *Erylus* | ERR1159473 | NCBI / SRA | [2] | Marine |
| *Erylus* | ERR1159474 | NCBI / SRA | [2] | Marine |
| *Eunapius* | [mgm4679569.3](https://www.mg-rast.org/mgmain.html?mgpage=overview&metagenome=mgm4679569.3) | MG-RAST | [1] | Freshwater |
| *Eunapius* | [mgm4679565.3](https://www.mg-rast.org/mgmain.html?mgpage=overview&metagenome=mgm4679565.3) | MG-RAST | [1] | Freshwater |
| *Eunapius* | [mgm4679570.3](https://www.mg-rast.org/mgmain.html?mgpage=overview&metagenome=mgm4679570.3) | MG-RAST | [1] | Freshwater |
| *Geodia* | ERR1159641 | NCBI / SRA | [2] | Marine |
| *Geodia* | ERR1159642 | NCBI / SRA | [2] | Marine |
| *Geodia* | ERR1159658 | NCBI / SRA | [2] | Marine |
| *Geodiidae* | ERR1158963 | NCBI / SRA | [2] | Marine |
| *Geodiidae* | ERR1159049 | NCBI / SRA | [2] | Marine |
| *Geodiidae* | ERR1159057 | NCBI / SRA | [2] | Marine |
| *Halichondria* | ERR1159650 | NCBI / SRA | [2] | Marine |
| *Halichondria* | ERR1159651 | NCBI / SRA | [2] | Marine |
| *Halichondria* | ERR1159663 | NCBI / SRA | [2] | Marine |
| *Hymedesmiidae* | ERR1159146 | NCBI / SRA | [2] | Marine |
| *Hymedesmiidae* | ERR1159147 | NCBI / SRA | [2] | Marine |
| *Hymedesmiidae* | ERR1159148 | NCBI / SRA | [2] | Marine |
| *Ianthella* | ERR1159844 | NCBI / SRA | [2] | Marine |
| *Ianthella* | ERR1159864 | NCBI / SRA | [2] | Marine |
| *Ianthella* | ERR1159865 | NCBI / SRA | [2] | Marine |
| *Ircinia* | ERR1158891 | NCBI / SRA | [2] | Marine |
| *Ircinia* | ERR1158949 | NCBI / SRA | [2] | Marine |
| *Ircinia* | ERR1158985 | NCBI / SRA | [2] | Marine |
| *Lithistida* | ERR1158892 | NCBI / SRA | [2] | Marine |
| *Lithistida* | ERR1158918 | NCBI / SRA | [2] | Marine |
| *Lithistida* | ERR1158946 | NCBI / SRA | [2] | Marine |
| *Mycalidae* | ERR1158890 | NCBI / SRA | [2] | Marine |
| *Mycalidae* | ERR1158968 | NCBI / SRA | [2] | Marine |
| *Mycalidae* | ERR1158969 | NCBI / SRA | [2] | Marine |
| *Niphates* | ERR1159436 | NCBI / SRA | [2] | Marine |
| *Niphates* | ERR1159437 | NCBI / SRA | [2] | Marine |
| *Petrosiidae* | ERR1158893 | NCBI / SRA | [2] | Marine |
| *Petrosiidae* | ERR1158920 | NCBI / SRA | [2] | Marine |
| *Petrosiidae* | ERR1158926 | NCBI / SRA | [2] | Marine |
| *Plakinidae* | ERR1158894 | NCBI / SRA | [2] | Marine |
| *Plakinidae* | ERR1158921 | NCBI / SRA | [2] | Marine |
| *Plakinidae* | ERR1159052 | NCBI / SRA | [2] | Marine |
| *Polymastia* | ERR1159582 | NCBI / SRA | [2] | Marine |
| *Polymastia* | ERR1159583 | NCBI / SRA | [2] | Marine |
| *Pseudoceratina* | ERR1159594 | NCBI / SRA | [2] | Marine |
| *Pseudoceratina* | ERR1159595 | NCBI / SRA | [2] | Marine |
| *Pseudoceratina* | ERR1159566 | NCBI / SRA | [2] | Marine |
| *Rhoaploeides* | ERR1159878 | NCBI / SRA | [2] | Marine |
| *Rhoaploeides* | ERR1159908 | NCBI / SRA | [2] | Marine |
| *Sarcotragus* | ERR1159090 | NCBI / SRA | [2] | Marine |
| *Sarcotragus* | ERR1159360 | NCBI / SRA | [2] | Marine |
| *Sarcotragus* | ERR1159367 | NCBI / SRA | [2] | Marine |
| *Sceptrulophora* | ERR1158919 | NCBI / SRA | [2] | Marine |
| *Sceptrulophora* | ERR1158932 | NCBI / SRA | [2] | Marine |
| *Sceptrulophora* | ERR1159347 | NCBI / SRA | [2] | Marine |
| *Stelletta* | ERR1159625 | NCBI / SRA | [2] | Marine |
| *Stelletta* | ERR1159626 | NCBI / SRA | [2] | Marine |
| *Stelletta* | ERR1159627 | NCBI / SRA | [2] | Marine |
| *Stylissa* | ERR1159331 | NCBI / SRA | [2] | Marine |
| *Stylissa* | ERR1159332 | NCBI / SRA | [2] | Marine |
| *Stylissa* | ERR1159334 | NCBI / SRA | [2] | Marine |
| *Theonella* | ERR1159622 | NCBI / SRA | [2] | Marine |
| *Theonella* | ERR1159624 | NCBI / SRA | [2] | Marine |
| *Xestospongia* | ERR1158956 | NCBI / SRA | [2] | Marine |
| *Xestospongia* | ERR1158987 | NCBI / SRA | [2] | Marine |
| *Xestospongia* | ERR1158988 | NCBI / SRA | [2] | Marine |

**References**

[1] Gaikwad, S., Shouche, Y.S., Gade, W.N. (2016). [Microbial community structure of two freshwater sponges using Illumina MiSeq sequencing revealed high microbial diversity.](https://www.ncbi.nlm.nih.gov/pubmed/27299740) AMB Express. 6, 40. doi: 10.1186/s13568-016-0211-2.

[2] [Thomas, T](https://www.ncbi.nlm.nih.gov/pubmed/?term=Thomas%20T%5BAuthor%5D&cauthor=true&cauthor_uid=27306690)., [Moitinho-Silva, L](https://www.ncbi.nlm.nih.gov/pubmed/?term=Moitinho-Silva%20L%5BAuthor%5D&cauthor=true&cauthor_uid=27306690)., [Lurgi, M](https://www.ncbi.nlm.nih.gov/pubmed/?term=Lurgi%20M%5BAuthor%5D&cauthor=true&cauthor_uid=27306690)., Björk, J.R., Easson, C., Astudillo-García, C., et al. (2016). Diversity, structure and convergent evolution of the global sponge microbiome. Nat. Commun. 7, 11870. doi: 10.1038/ncomms11870.
