# Supplementary material for: Freshwater Sponge Tubella variabilis Presents Richer Microbiota Than Marine Sponge Species
Source: Front Microbiol. 2019 Dec 3;10:2799. doi: 10.3389/fmicb.2019.02799 (PMC6902092; doi:10.3389/fmicb.2019.02799)
Supplement: TABLE S2 — Antimicrobial activity assay performed with bacteria isolated from sponges and freshwater against Staphylococcus aureus ATCC29213. [file Table_2.DOCX]

| **Table S2.** Antimicrobial activity assay performed with bacteria isolated from sponges and freshwater against *Staphylococcus aureus* ATCC29213. | | | | |
| --- | --- | --- | --- | --- |
| **Sponge isolates** | **GenBank accession number** | **Inhibition zone (diameter*)** |  |  |
| *Aquitalea* 10xC2-9 | MH477677.1 | 11 mm |  |  |
| *Aquitalea* RA3-8 | MH426925.1 | 11 mm |  |  |
| *Chromobacterium* CA1-5 | MH426927.1 | 17 mm |  |  |
| *Dickeya* BB2-9 | MH426873.1 | 20 mm |  |  |
| *Enterobacter* BB1-12 | MH454639.1 | 16 mm |  |  |
| *Enterobacteriaceae* 10xA1-5a | MH477676.1 | 11 mm |  |  |
| *Enterobacteriaceae* BA1-4 | MH477682.1 | 11 mm |  |  |
| *Enterobacteriaceae* BA2-1 | MH477684.1 | 11 mm |  |  |
| *Enterobacteriaceae* BB2-6 | MH454629.1 | 13 mm |  |  |
| *Enterobacteriaceae* MB3-3 | MH426901.1 | 21 mm |  |  |
| *Fictibacillus* RC2-5 | MH426921.1 | 11 mm |  |  |
| *Klebsiella* 10xA2-5 | MH477674.1 | 10 mm |  |  |
| *Klebsiella* BA1-5 | MH477683.1 | 8 mm |  |  |
| *Klebsiella* BB1-1 | MH477689.1 | 7 mm |  |  |
| *Klebsiella* BB2-10 | MH424480.1 | 15 mm |  |  |
| *Klebsiella* BB2-2 | MH477691.1 | 10 mm |  |  |
| *Klebsiella* BB2-3 | MH454637.1 | 11 mm |  |  |
| *Klebsiella* BB2-5 | MH477693.1 | 9 mm |  |  |
| *Klebsiella* CB2-2 | MH426908.1 | 10 mm |  |  |
| *Lactococcus* BB2-7 | MH426874.1 | 9 mm |  |  |
| *Staphylococcus* RA2-9 | MH426924.1 | 10 mm |  |  |
| **Freshwater isolates** |  |  |  |  |
| *Chryseobacterium* MW3-7 | MH426884.1 | 10 mm |  |  |
| *Enterobacter* BW3-11 | MH424476.1 | 7 mm |  |  |
| *Enterobacter* BW3-6 | MH426882.1 | 7 mm |  |  |
| *Klebsiella* 10xW2-13 | MH470400.1 | 13 mm |  |  |
| *Klebsiella* BW2-13 | MH426881.1 | 18 mm |  |  |
| *Klebsiella* CW2-2 | MH426858.1 | 11 mm |  |  |
| *Pseudomonas* 10xW2-3 | MH470396.1 | 30 mm |  |  |
| *Pseudomonas* BW1-3 | MH424486.1 | 28 mm |  |  |
| *Pseudomonas* RW1-13 | MH426919.1 | 30 mm |  |  |
| *Pseudomonas* RW1-7 | MH426915.1 | 22 mm |  |  |
| *Pseudomonas* RW1-8 | MH426914.1 | 12 mm |  |  |
| *Stenotrophomonas* BW2-11 | MH426883.1 | 15 mm |  |  |
| *Stenotrophomonas* BW2-12 | MH424483.1 | 15 mm |  |  |
| *Stenotrophomonas* BW3-4 | MH426878.1 | 7 mm |  |  |
| *Stenotrophomonas* BW3-9 | MH426879.1 | 10 mm |  |  |
| *Average of three measures of the diameter of inhibition zone of *S. aureus* growth. | | | |  |
|  |  |  |  |  |
|  |  |  |  |  |
